# Effects of Temperature on Auditory Sensitivity in Eurythermal Fishes: Common Carp *Cyprinus carpio* (Family Cyprinidae) versus Wels Catfish *Silurus glanis* (Family Siluridae)

**DOI:** 10.1371/journal.pone.0108583

**Published:** 2014-09-25

**Authors:** Isabelle Pia Maiditsch, Friedrich Ladich

**Affiliations:** Department of Behavioural Biology, University of Vienna, Vienna, Austria; Universität Bielefeld, Germany

## Abstract

**Background:**

In ectothermal animals such as fish, -temperature affects physiological and metabolic processes. This includes sensory organs such as the auditory system. The reported effects of temperature on hearing in eurythermal otophysines are contradictory. We therefore investigated the effect on the auditory system in species representing two different orders.

**Methodology/Principal Findings:**

Hearing sensitivity was determined using the auditory evoked potentials (AEP) recording technique. Auditory sensitivity and latency in response to clicks were measured in the common carp *Cyprinus carpio* (order Cypriniformes) and the Wels catfish *Silurus glanis* (order Siluriformes) after acclimating fish for at least three weeks to two different water temperatures (15°C, 25°C and again 15°C). Hearing sensitivity increased with temperature in both species. Best hearing was detected between 0.3 and 1 kHz at both temperatures. The maximum increase occurred at 0.8 kHz (7.8 dB) in *C. carpio* and at 0.5 kHz (10.3 dB) in *S. glanis*. The improvement differed between species and was in particular more pronounced in the catfish at 4 kHz. The latency in response to single clicks was measured from the onset of the sound stimulus to the most constant positive peak of the AEP. The latency decreased at the higher temperature in both species by 0.37 ms on average.

**Conclusions/Significance:**

The current study shows that higher temperature improves hearing (lower thresholds, shorter latencies) in eurythermal species from different orders of otophysines. Differences in threshold shifts between eurythermal species seem to reflect differences in absolute sensitivity at higher frequencies and they furthermore indicate differences to stenothermal (tropical) species.

## Introduction

Physiological and metabolic processes of ectothermal animals are affected in any environment that is characterized by major and rapid temperature changes. Many fish species occur in habitats in which the water temperature changes either quickly such as in shallow waters or slowly or not at all such as in oceans or deep lakes. Fishes can also experience rapid temperature changes when moving to different water depths, or slower changes with season, during which the acclimation time is longer [1], [2]. Ambient temperature affects the speed of metabolic and physiological processes such as respiration, the immune system or growth [3], [4], [5]. Furthermore, temperature affects the behaviour including the locomotory activity [1], [6], [7].

Ambient temperature is also known to affect the sensitivity of sensory systems such as the lateral line [8] and the auditory system. Influences of temperature on the auditory system have been studied in many ectothermal taxa such as insects [9], [10] amphibians [11], [12], [13] and reptiles [14], [15]. In general, a decrease in body temperature results in a decline in auditory sensitivity.

Fish rely on sound production and hearing for orientation, intraspecific communication or prey and predator detection [16], [17], [18], [19], [20]. A few studies described influences of ambient temperature on sound characteristics and hearing sensitivity in fish. The sound duration and the fundamental frequency tend to increase with temperature, whereas the pulse period decreased [21], [22], [23], [24], [25], [26].

Dudok van Heel [27] observed that the detectable frequency range broadened with increasing temperature in the European minnow *Phoxinus phoxinus*. Fay and Ream [28] showed that higher temperature boosts the spontaneous activity and sensitivity in the auditory neuron of the goldfish *Carassius auratus*. Auditory sensitivity decreased at lower temperature within hours in the walleye pollock *Theragra chalcogramma* [29]. Among catfishes, Wysocki et al. [2] investigated the effects of temperature on hearing in the eurythermal channel catfish *Ictalurus punctatus* and the stenothermal pictus catfish *Pimelodus pictus*, and Papes and Ladich [26] conducted similar experiments on the Striped Raphael Catfish *Platydoras armatulus*Interestingly, thresholds shifts seem to be more pronounced in the eury- than in the stenothermal catfish species from the Amazonian river system.

Temperatures affect hearing thresholds as well as the resolution of temporal patterns of acoustic information in ectothermal animals. Interpeak latencies increased in anurans when the temperature dropped below 20°C [30]. The influence of ambient water temperature on temporal processing and latencies in fish has been demonstrated by Papes and Ladich [26] in the thorny catfish *P. armatulus*. Wysocki and Ladich [31] showed that representatives of several fish families were able to detect pulse periods of less than 2 ms. This ability enables fish to detect the temporal resolution of conspecific sounds [32], [33].

The goal of this study was to investigate the effect of ambient temperature on the auditory system of two eurythermal species representing different otophysan orders. It was designed to answer the questions how eurythermal species are affected by temperature and how this differs compared to stenothermal (tropical) species. The Common carp *Cyprinus carpio* (order Cyrpiniformes)and the Wels catfish *Silurus glanis* (order Siluriformes) were chosen because they represent two different orders and because they possess improved hearing abilities (due to peripheral hearing structures), which are more likely affected by temperature changes [2]. Both species inhabit freshwaters in Eurasia and can survive under a wide range of temperatures from 0°C to 30°C [34], [35], [36], [37]. We also investigated the change in latencies in response to single-click stimuli and thus temporal processing of acoustic signals in both species at different temperatures.

## Materials and Methods

### Ethics statement

Experiments were performed with permission of the Austrian Federal Ministry of Science and Research (GZ 66.006/0023-II/10b/2008).

### Animals

Nine Common carps, *Cyprinus carpio* Linnaeus 1758 [11.3−12.8 cm standard length (SL) 40−64 g body mass (BM)] and eight Wels catfish, *Silurus glanis* Linnaeus 1758 [23.0−30.6 cm SL, 103−211 g BM] were used for his study. *S. glanis* were obtained from a fish hatchery (Fischzucht Pottenbrunn, Pottenbrunn, Austria), *C. carpio* from a private fish pond near Vienna.

Fish were kept in glass tanks (110×55×30 cm or 100×50×50 cm) with a sand bottom equipped with plastic tubes, roots and artificial plants. External filters were used and a 12 h: 12 h L:D cycle was maintained. *S. glanis* were fed frozen food (chironomid larvae) and *C. carpio* were fed commercially prepared food (Tetra Pond; www.tetra-fish.com) as well as frozen food (chironomid larvae). The baseline temperature was 20±1°C.

The fishes were acclimated to the baseline temperature (20°C) for more than one month before experiments started. The temperature in the holding tanks was controlled using a cooling system (Hailea HC-300A and HC-130A; Guangdong Heilea Group CO., Ltd.) and submersible heaters. Temperature was controlled daily. The temperature of the holding water was changed at a rate of 1°C per day until the test temperature of 15°C or 25°C was reached. Fish had an acclimation time of at least three weeks to each experimental temperature before hearing measurements started. First, fish were acclimated and measured at 15°C, followed by 25°C and finally again at 15°C for control purposes. Fish had more than three weeks rest after each hearing test.

At each water temperature the audiograms of eight *S. glanis* and nine *C. carpio* were measured. In *S. glanis*, individuals were recognized based on different colour patterns. Individuals of *C. carpio* were marked on their fins.

### Auditory sensitivity measurements

Auditory sensitivity was measured using the auditory evoked potential (AEP) recording technique [38], [39], [40].

The test subjects were immobilized during the hearing test using Flaxedil (gallamine triethiodide; Sigma-Aldrich, Vienna, Austria). The dosage used was 9−24 µg g ^−1^ for *S. glanis* and 4−7 µg g ^−1^ for *C. carpio* and allowed the fish to breath during the experiment. A respiration pipette was inserted into the animals’ mouth. Respiration was achieved by a temperature-controlled gravity-fed circulation system.

The fish were secured in a plastic tub (45×35 cm; 18 cm height). The bottom was covered with fine sand. The temperature was maintained at either 15±1°C or 25±1°C using cooling packs or a submersible heater. The fish’s head was positioned just below the water surface. The plastic tub was positioned on an air table (TCM Micro-g 63-540), which rested on a vibration-isolated concrete plate. The entire setup was enclosed in a soundproof room constructed as a Faraday cage (interior dimensions: 3.2×3.2×2.4 m).

For AEP recordings, silver electrodes (0.32 mm diameter) were placed in the midline of the skull. The recording electrode was positioned over the region of the medulla and the reference electrode cranially between the nares; both were pressed firmly against the skin, which was covered with a small piece of Kimwipes tissue paper to keep it moist, in order to ensure proper contact during experiments. Shielded electrodes leads were attached to the differential input of a preamplifier (Grass P-55, Grass Instruments, West Warwick, RI, USA; gain 10,000x, high-pass at 30 Hz, low-pass at 1 kHz). A ground electrode was placed in the water. Stimuli presentation and AEP-waveform recording were specified using a modular rackmount system (TDT System 3, Tucker-Davis Technologies, Gainesville, FL, USA) running TDT BioSig RP Software.

### Sound stimuli

Sound stimuli were generated using TDT SigGen RP software and fed through a power amplifier (Alesis RA 300, Alesis Corporation, Los Angeles, CA, USA) to a dual-cone speaker (Tannoy System 600, frequency response 50 Hz to 15 kHz±3dB), which was placed 1 m above the tub. Sound stimuli were presented as tone bursts at a repetition rate of 21 per second.

Hearing thresholds were determined at frequencies of 0.1, 0.3, 0.5, 0.8, 1, 2 and 4 kHz, presented in random order. Rise and fall times were one cycle at 0.1 and 0.2 kHz, and two cycles at all other frequencies. All bursts were gated using a Blackman window. The stimuli were presented at opposite polarities (180° phase shifted) for each test condition and the corresponding AEPs were averaged by the BioSig RP software in order to eliminate stimulus artefacts. The sound pressure level (SPL) of tone-burst stimuli was reduced in 4 dB steps until the AEP waveform was no longer apparent. The lowest SPL for which a repeatable AEP trace could be obtained, which was determined by overlaying replicate traces, was considered the threshold. A hydrophone (Brüel & Kjaer 8101) was positioned near the right side of each fish (2 cm apart) to determine absolute SPLs values underwater close to the subjects. The absolute SPL was determined by analyzing the hydrophone recording at the threshold. Using Bio-Sig RP, the RMS voltage of the largest (i.e., center) sinusoid of a particular tone-burst recording was determined. This RMS voltage was then used to calculate the absolute SPL re 1 µPa based on the sensitivity of the hydrophone and the amplification factor of the hydrophone amplifier and of the TDT system.

### Latency measurements

**Figure 1:**
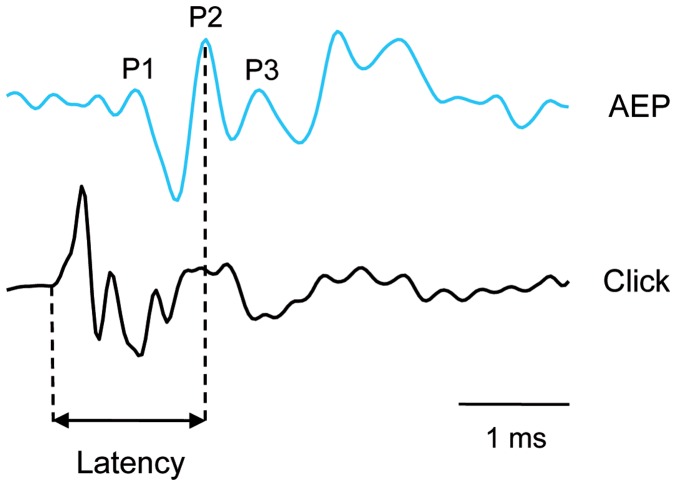
Oscillograms of an AEP and a click stimulus. AEP of a *S. glanis* in response to the click stimulus presented 28 dB above hearing threshold. The first, second and third positive peak (P1, P2, P3) are labelled. The double-headed arrow indicates the latency measured from the onset of the click stimulus to the second positive peak (P2).

Latency measurements followed the method described by Wysocki and Ladich [31] and Papes and Ladich [26]. AEPs in response to a single click consisted of a series of negative and positive deflections. The positive AEP peaks were denominated with P for positive peaks (directed upwards) by ascending numbers. The latency was defined as time between the onset of the click stimulus and the most constant prominent peak of the AEP (P2) found in responses to this click stimulus in all individuals (Fig. 1).

The single click was presented 28 dB above hearing threshold in both species. Clicks were generated and presented using the TDT SigGen RP software. They were fed through a RP 2.1 realtime processor, a PA5 programmable attenuator, and a power amplifier (Alesis RA 300) to the air speaker (Tannoy System 600). Single clicks were presented to the animals at a repetition rate of 35 per second.

### Statistical Analyses

All data were tested for normal distribution using the Kolmogorov-Smirnov-test, and when data were normally distributed, parametric statistical tests were applied. Audiograms obtained at three temperatures (15°C, 25°C and 15°C repeated) were compared by a two-factorial analysis of variance (ANOVA) using a general linear model where one factor was temperature and the other was frequency. The temperature factor alone should indicate overall differences in sensitivity between temperatures (and in combination with the frequency factor if different tendencies exist at different frequencies of the audiogram)s. A repeated measures ANOVA followed by a Bonferroni post hoc tests was calculated to determine differences between thresholds at each frequency. Differences between latencies were calculated using a Friedman-test followed by a Wilcoxon test. All statistical tests were run using SPSS 17.0. The significance level was set at p≤0.05.

## Results

### Auditory sensitivities

#### Cyprinus carpio

**Figure 2:**
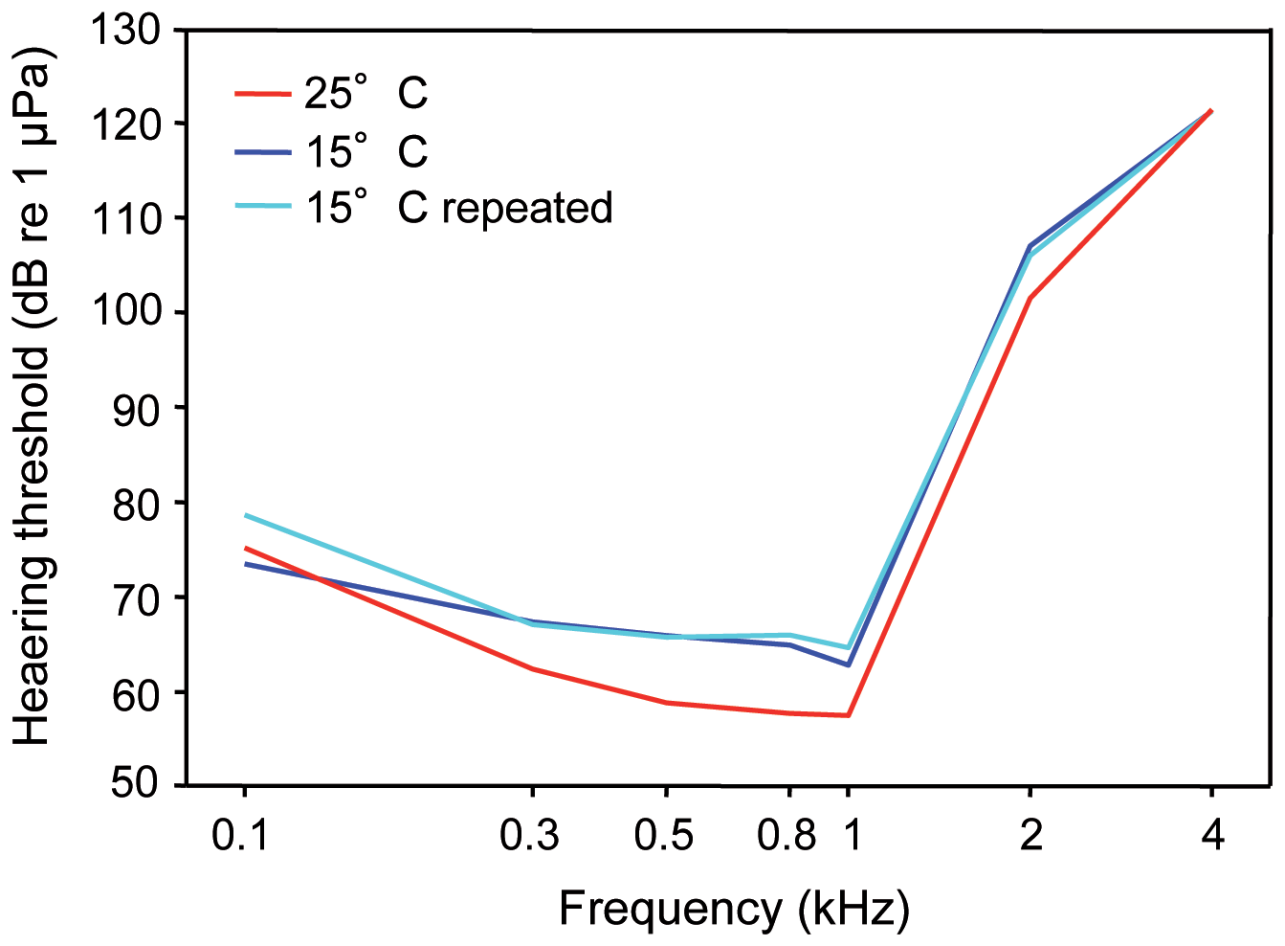
Auditory evoked potential audiogram at 15°C and 25°C. Mean hearing thresholds of *C. carpio* kept 15°C, 25°C and 15°C repeated. N = 9.

Hearing curves showed best hearing between 0.3 and 1 kHz at both temperatures and a rapid decline towards higher frequencies. A two-factorial ANOVA revealed that the auditory sensitivity was significantly lower at 15°C (F _2,168_ = 36.9, p≤0.001) and that there was a significant interaction between temperature and frequency (F _12,168_ = 3.05, p≤0.001). Thus, changes in auditory sensitivity showed different trends at different frequencies (Table 1, Fig. 2). A Bonferroni post-hoc test showed no significant difference between both 15°C audiograms, but significance between both 15°C and 25°C (15°C vs. 25°C: p≤0.001; 25°C vs. 15°C repeated: p≤0.001; 15°C vs. 15°C repeated: n.s.).

**Table 1:** Mean (± S.E.) hearing thresholds of *C. carpio* measured at 15°C, 25°C and 15°C repeated. N = 9.

Frequency kHz	15°C dB re 1 µPa	25°C dB re 1 µPa	15°C repeated dB re 1 µPa
**0.1**	73.4±1.3	75.1±1.1	78.7±1.3
**0.3**	67.3±1.0	62.3±1.2	67.1±1.1
**0.5**	65.9±0.9	58.8±1.1	65.8±0.6
**0.8**	64.9±1.1	57.7±1.6	66±1.2
**1**	62.8±1.3	57.4±0.6	64.7±0.7
**2**	107±2.0	101.4±1.7	106±1.1
**4**	121.2±1.3	121.3±0.7	121.2±0.5

#### Silurus glanis

**Figure 3:**
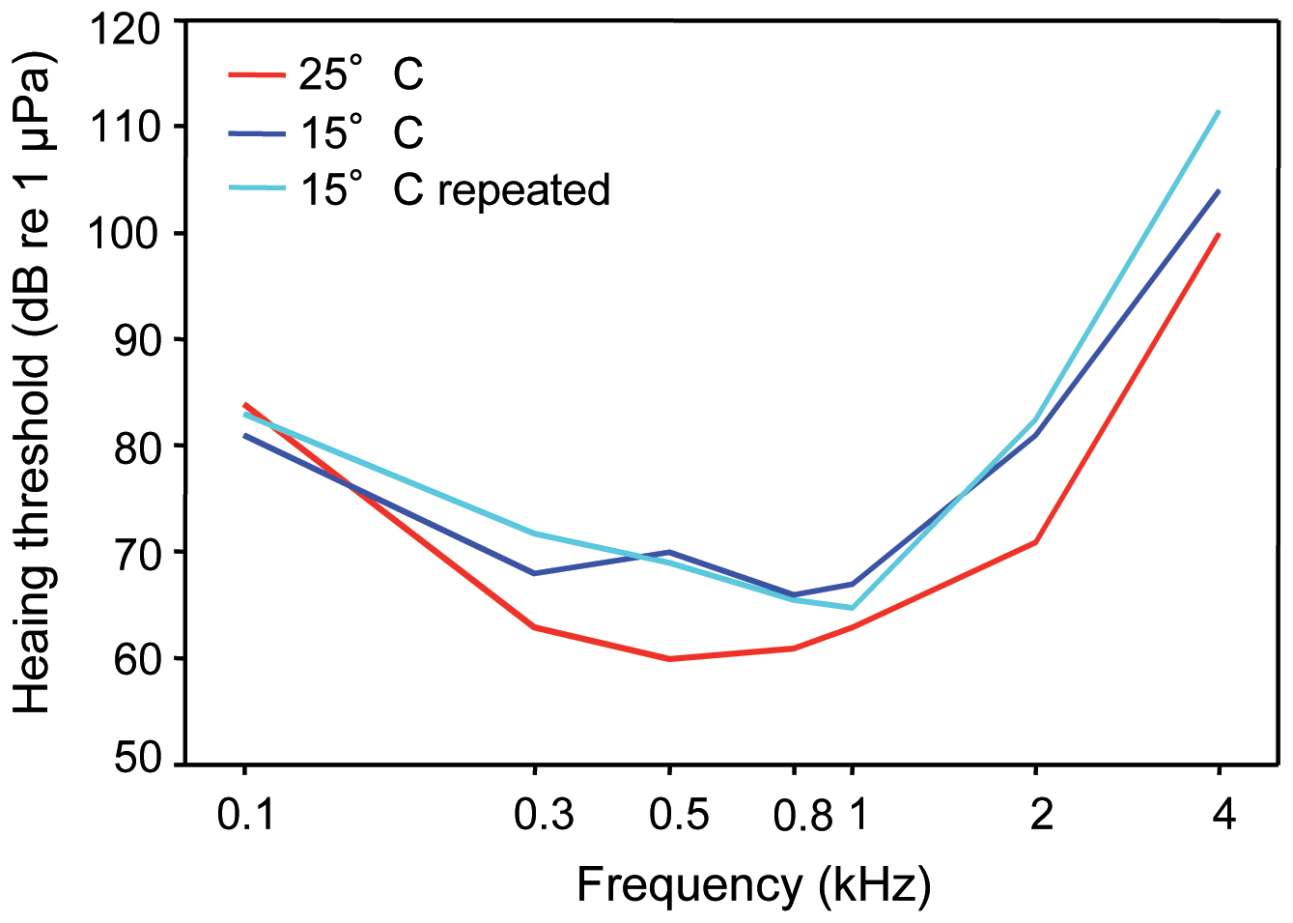
Auditory evoked potential audiogram at 15°C and 25°C. Mean hearing thresholds of *S. glanis* kept at 15°C, 25°C and 15°C repeated. N = 8.

Hearing sensitivity curves showed best hearing between 0.3 and 1 kHz and a decline towards 4 kHz. Auditory sensitivities were significantly lower at the lower temperatures, as revealed by a two-factorial ANOVA (F _2,176_ = 346.6, p<0.001), and there was a significant interaction between temperature and frequency (F _7,176_ = 4.313, p≤0.001). Therefore, changes in auditory sensitivity showed different trends at different frequencies (Table 2, Fig. 3).

**Table 2:** Mean (± S.E.) hearing thresholds of *S. glanis* measured at 15°C, 25°C and 15°C repeated. N = 8.

Frequency kHz	15°C dB re 1 µPa	25°C dB re 1 µPa	15°C repeated dB re 1 µPa
**0.1**	81.4±1.8	83.9±1.3	83±1.6
**0.3**	67.9±1.9	62.9±0.8	71.8±0.9
**0.5**	70.4±2.3	60±0.7	69±0.6
**0.8**	65.9±1.6	60.9±1.3	65.5±0.7
**1**	66.9±1.7	62.5±1.3	64.8±0.5
**2**	80.9±1.6	71.4±2.3	82.5±1.2
**4**	104±1.1	100.4±1.7	111.5±1.4

Catfish had better hearing sensitivity at the higher temperature, especially above 300 Hz (Fig. 3). A Bonferroni post-hoc test showed a significant difference between 25°C and both 15°C audiograms but no difference between both 15°C audiograms (15°C vs. 25°C: p≤0.001; 25°C vs. 15°C repeated: p≤0.001; 15°C vs. 15°C repeated: n.s.).

#### Comparison between *C. carpio* and *S. glanis*

Both *C. carpio* and *S. glanis* showed no change in sensitivity at the lowest frequency measured (100 Hz) when the temperature increased from 15°C to 25°C (Repeated measures ANOVA). A significant increase was found at higher frequencies except for 4 kHz in the carp. In *C. carpio* the main change in sensitivity was observed between 0.3 and 2 kHz, whereas in the *S. glanis* changes of more than 5 dB were found up to 4 kHz (Table 3). The maximum increase occurred at 0.8 kHz (7.8 dB) in *C. carpio* and at 0.5 kHz (10.3 dB) in *S. glanis.*

**Table 3:** Mean differences in hearing sensitivity of *C. carpio* and *S. glanis,* between the two tested temperatures (mean of 15°C and 15°C repeated) and 25°C.

Frequency kHz	*S. glanis* dB	*C. carpio* dB	Difference dB
**0.1**	1.7	0.9	0.8
**0.3**	6.9	4.9	2
**0.5**	9.7	7.1	2.6
**0.8**	4.8	7.8	−3
**1**	3.4	6.3	−2.9 *
**2**	10.3	5.1	5.2 *
**4**	7.4	0.1	7.3 *

The last column gives the difference in threshold changes between *S. glanis* and *C. carpio*. Asterisks indicate significant differences between species.

A two-factorial ANOVA revealed that the improvement in hearing differed between the two species (F _1,105_ = 6.35, p<0.05) and that there was an interaction between the difference and the frequency (F _6,105_ = 6.72, p<0.001). Hearing sensitivity improved to a higher degree in *C. carpio* at 1 and in *S. glanis* at 2 and 4 kHz (Table 3).

### Latencies in response to single clicks

#### Cyprinus carpio

**Figure 4:**
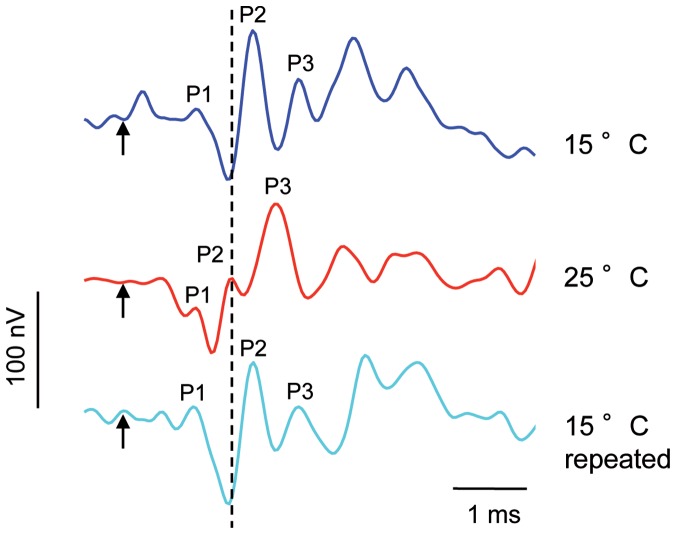
AEPs of one specimen of *C. carpio* in response to a single-click stimulus. Click stimulus presented 28 dB above hearing thresholds at both temperatures. Arrows indicate onset. The vertical dashed line indicates the position of the P2 peak at 25°C relative to the P2 peak at 15°C and 15°C repeated.

AEP waveforms of *C. carpio* in response to a single-clicks consisted of a series of positive and negative deflections. AEPs started with a positive peak, followed by a negative peak at all three tested temperatures (Fig. 4). In this study, the main constant positive peak (P2) of the AEPs was analyzed.

**Figure 5:**
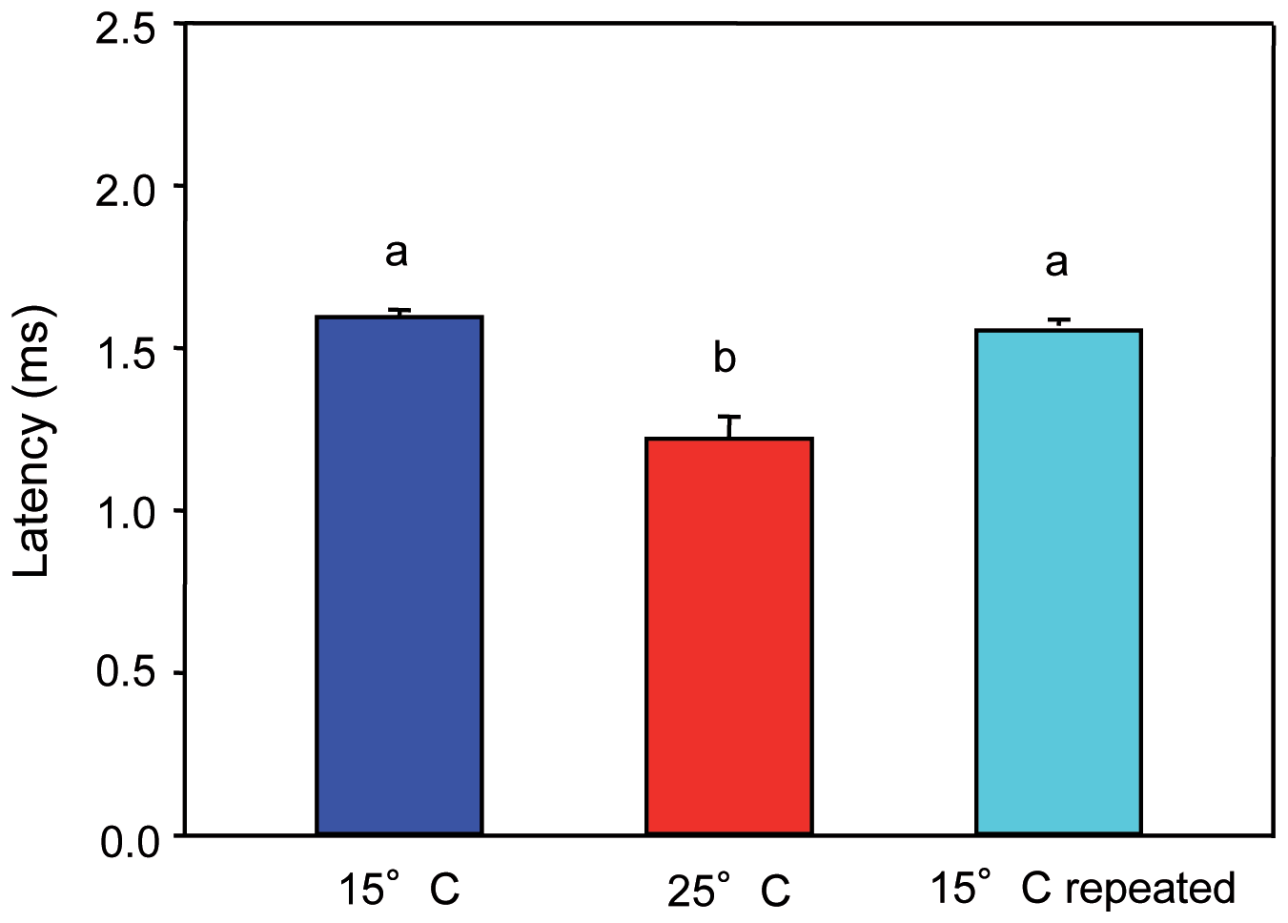
Latency between the onset of the click and the second positive peak. Mean (+ S.E.) latency of *C. carpio* kept at 15°C, 25°C and 15°C repeated. N = 9. Different letters (a, b) indicate significant differences between temperatures (p≤0.05).

The latency between the onset of the single-click stimulus and P2 differed between temperatures in *C. carpio* (Friedman test: χ^2^ = 10.34, df = 2, p≤0.01). Wilcoxon tests showed that the delay in the onset of P2 was significantly longer at lower temperature and that there was no significant difference between latencies in both 15°C tests (15°C vs. 25°C: p≤0.05; 25°C vs. 15°C repeated: p≤0.05; 15°C vs. 15°C repeated: n.s.) (Table 4, Fig. 5).

**Table 4:** Mean (± S.E.) latency of the second positive peaks (P2) of *C. carpio* and *S. glanis* measured at 15°C, 25°C and 15°C repeated calculated as the time period between the onset of a single click stimulus and the second positive peak.

Temperature	Latency (ms) *C. carpio*	Latency (ms) *S. glanis*
**15°C**	1.59±0.02	2.02±0.2
**25°C**	1.22±0.1	1.63±0.03
**15°C repeated**	1.55±0.02	2.02±0.2

#### Silurus glanis

**Figure 6:**
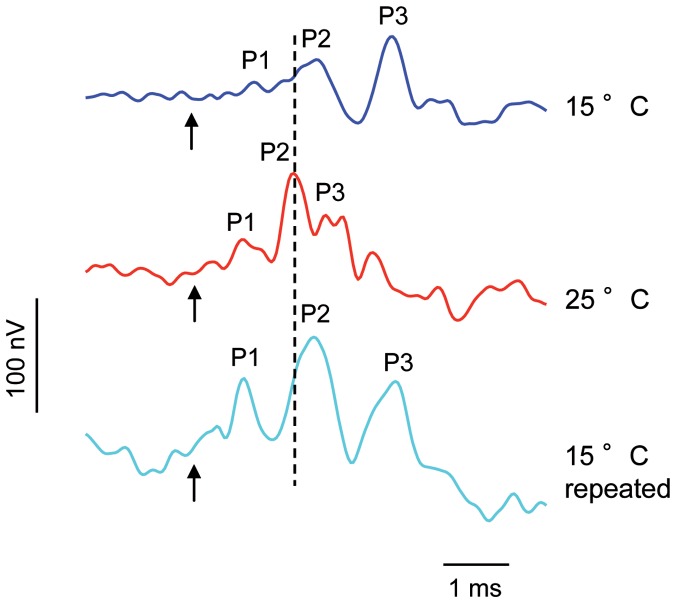
AEPs of one specimen of *S. glanis* in response to a single-click stimulus. Click stimulus was presented 28 dB above hearing thresholds at both temperatures. Arrows indicate onset of the single-click stimulus. The vertical dashed line indicates the position of the P2 peak at 25°C relative to the P2 peak at 15°C and 15°C repeated.

**Figure 7:**
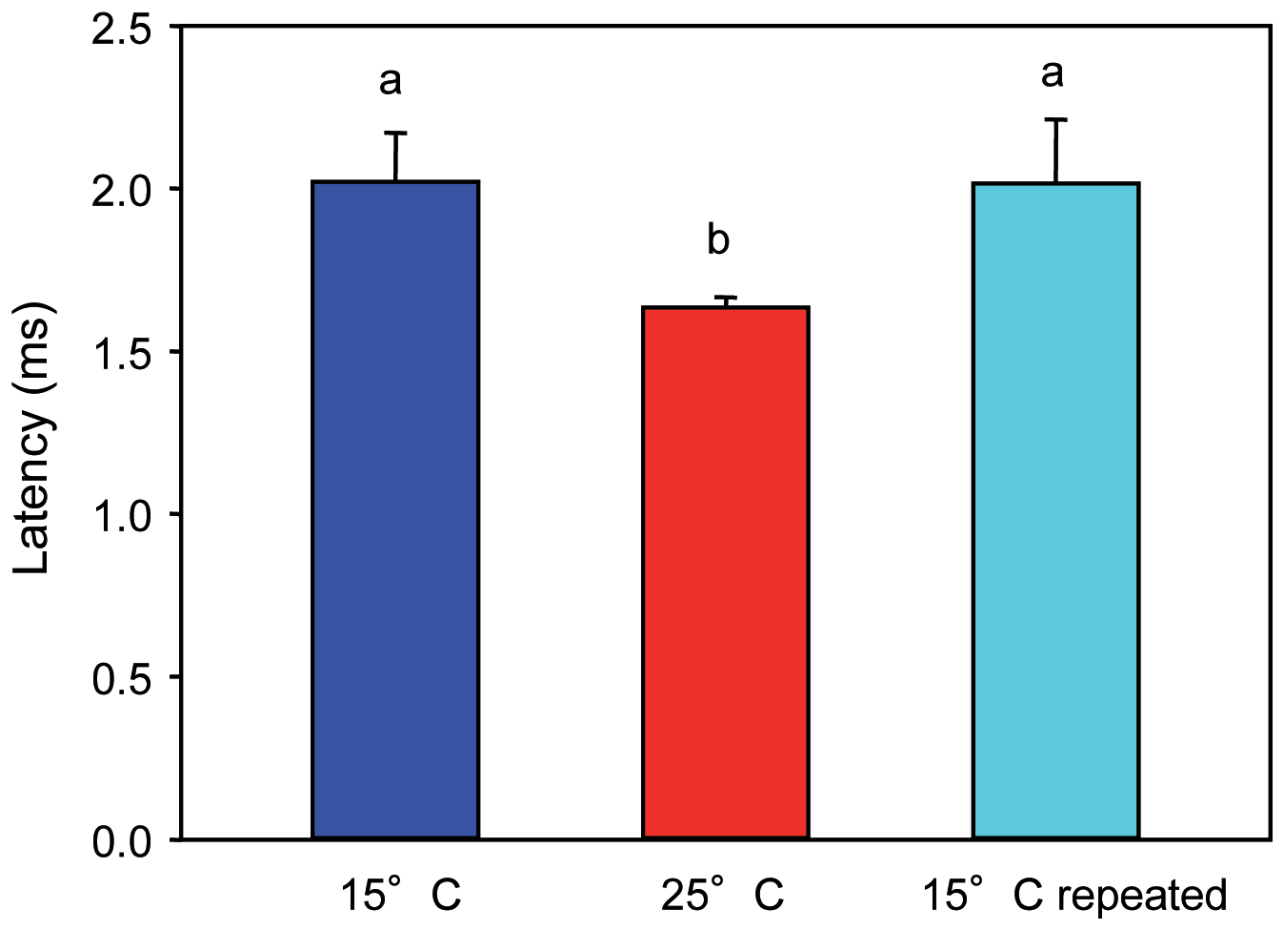
Latency between the onset of the click and the second positive peak. Mean (+ S.E.) latency of *S. glanis* kept at 15°C, 25°C and 15°C repeated. N = 8. Different letters (a, b) indicate significant differences between temperatures (p≤0.05).

In *S. glanis*, similar to *C. carpio*, the AEP in response to a single-click stimulus consisted of a series of positive and negative peaks (Fig. 6). The delay between the onset of the single-click stimulus and the first constant prominent peak (P2) was similar in both 15°C tests and shorter at 25°C (Friedman test: χ^2^ = 7.45, df = 2, p≤0.05). A Wilcoxon test showed that the delay in the onset of P2 was significantly longer at lower temperature and that there was no significant difference between both 15°C measurements (15°C vs. 25°C: p≤0.05; 25°C vs. 15°C repeated: p≤0.05; 15°C vs. 15°C repeated: n.s.) (Table 4, Fig. 7).

## Discussion

Hearing sensitivity in *C. carpio* as well as *S. glanis* is significantly higher at higher water temperatures, but the change in sensitivity differs between species. Temperature dependence of hearing sensitivity has been described in ectothermal animals besides fish such as in insects, amphibians and reptiles. In insects, the most sensitive hearing frequency, the spike rate and sensitivity increases [9], [41], [42]. Similar increases in hearing capability with temperature were shown in amphibians [11], [43] and reptiles [44].

Effects of ambient temperature on the auditory system have been shown in several fish species [2], [26], [27], [29], but the results of these studies vary. Dudok van Heel [27] showed that the detectable frequency range became wider in the European minnow, but he did not mention any change in absolute sensitivity. At higher temperature, the upper limit of frequency discrimination shifted in the minnow from 1200 Hz up to 1600 Hz. In a single specimen of the walleye pollock (*Theragra chalcogramma*), the hearing thresholds decreased by 8 dB at 350 Hz when temperature rose by 8° [29]. No acclimation periods were reported in the prior studies. Detailed studies involving at least three-week acclimation periods to different temperatures have only been conducted in otophysines so far.

### Temperature effects on hearing sensitivity in eurythermal fish

**Figure 8:**
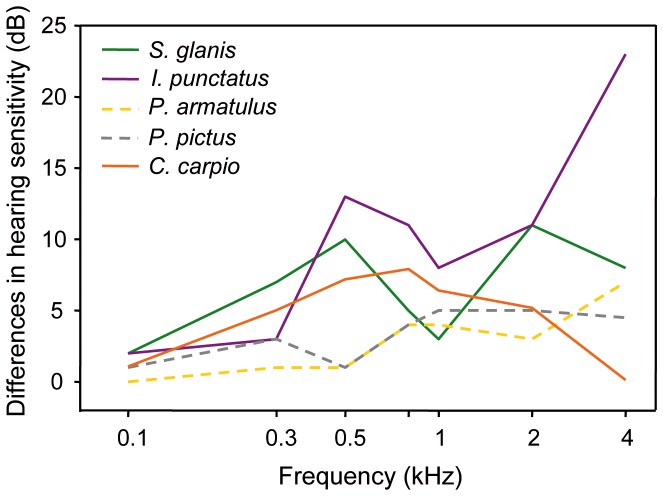
Comparison of the changes in hearing sensitivity in eurythermal (solid lines) and stenothermal (dashed lines) otophysines. *C. carpio* and *S. glanis* (recent study, 15°C vs. 25°C), *I. punctatus* (Wysocki et al., 2009: 18°C vs. 26°C), Amazonian catfishes *P. pictus* (Wysocki et al., 2009: 22°C vs. 30°C) and *P. armatulus* (Papes and Ladich, 2011; 22°C vs. 30°C).

Prior to this study, temperature effects on auditory sensitivity have been studied in detail in only one eurythermal fish. Eurythermal species can tolerate major changes in ambient water temperature. Wysocki et al. [2] observed major shifts in hearing thresholds in the North American channel catfish *I. punctatus* after acclimation to different temperatures. Changes in hearing sensitivity occurred especially at higher frequencies. Auditory sensitivity increased by 36 dB at 4 kHz when the temperature was raised from 10°C to 26°C. Here we compare the changes in sensitivity observed between 18°C and 26°C in *I. punctatus,* with those found in the present study in *C. carpio* und *S. glanis* between 15°C and 25°C. The two eurythermal catfish species studied, namely the channel catfish [2] and the European wels, showed a frequency-dependent increase in hearing sensitivity with increasing temperature. This trend was more pronounced in *I. punctatus*, whose sensitivity increased by 23 dB at 4 kHz (versus only 7 dB in *S. glanis*) (Fig. 8).

Interestingly, the temperature-dependent increase in sensitivity differed between both catfish species and the cypriniform, especially at 4 kHz. This points to major differences between otophysine families and orders. In fact *C. carpio* is less sensitive at 4 kHz than both catfish species with large unpaired swim bladders (25°C: 121 dB in *C. carpio* versus 100 dB in *S. glanis* and 81 dB in *I. punctatus*) [2]. The low sensitivity of *C. carpio* at 4 kHz is apparently unaffected by temperature. Moreover, the high sensitivity of *I. punctatus* at 4 kHz is affected much more than the lower sensitivity in *S. glanis* (23 dB change versus 7 dB). Thus, the differences in threshold shifts between eurythermal otophysines probably reflects the difference in the absolute sensitivity at higher frequencies.

Changes in ambient water temperature affect the auditory system of all eurythermal species investigated so far, although the degree of the sensitivity change differs considerably between species. Wysocki et al. [2] expected small changes in hearing in eurythermal fish species because they are adapted to a wide range of temperature in their habitats, which should lead to more resistance to temperature changes. Even so, differences were found in hearing ability between temperatures among all three species, especially at higher frequencies. Reasons could be that lower temperatures affect the auditory system more than higher ones and that fish are able to gain heat tolerance more rapidly than cold tolerance [2], [45].

### Comparison between stenothermal and eurythermal fish

Stenothermal species live in habitats characterized by small fluctuations in ambient temperatures (tropical regions) and should tolerate only small temperature changes. Wysocki et al. [2] and Papes and Ladich [26] observed that a temperature increase of 8°C (from 22°C to 30°C) resulted in only a small sensitivity improvement in two Amazonian catfish species from two different families. Both the pimelodid *Pimelodus pictus* and the doradid *Platydoras armatulus* showed a similar increase in hearing sensitivity of up to 5 dB (Fig. 8) even though the hearing curve is U-shaped in *P. armatulus* and ramp-like in *P. pictus*.

*Cyprinus carpio* showed smaller changes in sensitivity, especially at higher frequencies, similar to the stenothermal species. This similarity is, as discussed above, probably due to the low absolute thresholds of the carp at higher frequencies (threshold at 4 kHz at 22°C: *P. pictus*: 73 dB re 1 µPa, *P. armatulus:* 84 dB).

The comparison between the eurythermal and stenothermal catfishes indicates that the influence of temperature on the auditory system may differ depending upon whether a species is physiologically adjusted to tolerate a wide or narrow temperature range [2]. Eurythermal catfish species seem to respond more to temperature changes than stenothermal species. Nonetheless, note that the temperature increase from 15 (18)°C to 25 (26)°C in eurythermal species cannot directly be compared to an increase from 22°C to 30°C in stenothermal species.

In summary, all otophysines investigated so far showed higher hearing sensitivity when temperature increased [2], [26], [46]. This finding agrees with results of other studies on fish and amphibians, showing that temperature changes affects the inner ear and the central auditory pathways. Fay and Ream [28] and Smotherman and Narins [47] already suggested that warming water temperatures increase the cell’s spontaneous activity, best frequency, as well as the cell’s sensitivity and responsiveness in the goldfish as well as electrical resonance for hair cell tuning in the leopard frog. None of the studies conducted in fish within the last decade reported an increase of the frequency range detectable with increasing temperature. Thus, Dudok von Heel’s [27] observation in the European minnow is not supported by recent studies.

### Latencies in response to clicks and temporal processing

The auditory system of fish species, particularly of otophysan fishes, is well adapted for temporal processing of acoustic stimuli [31], [32], [33], [48]. In the current study, the latency between the onset of a single-click stimulus and the second positive peak of the AEP decreased similarly in *C. carpio* and *S. glanis* when the temperature increased. Latencies became shorter by approximately 0.35 ms in *C. carpio* and 0.39 ms in *S. glanis*. A comparison of the P2 latencies between *C. carpio* and *S. glanis* revealed that the carp’s P2 showed up faster by approximately 0.45 ms at 15°C and 0.41 ms at 25°C. This faster response to click stimuli in carps could point to differences in the temporal processing of short acoustic stimuli between otophysan families. The different latencies could furthermore reflect different auditory pathways between both species, indicating that P2s are generated in different brainstem nuclei [49]. Finally, the longer latencies in catfish could be because the tested catfish were twice as long as carps, resulting in longer distances between the swim bladder and the inner ear and in longer auditory pathways in the catfish (standard length: 11–13 cm in carps versus 23–31 cm in catfish).

A similar trend toward shorter latencies at higher temperatures has been found in the stenothermal doradid *P. armatulus.* Papes and Ladich [26] showed that the latency decreased in three out of four AEP peaks (P1, N2 and P2) at the higher temperature. However, a direct comparison of latencies between the silurid and the doradid catfish is not possible because AEP waveforms differ considerably between species and it is unclear if P2s are generated by the same brainstem nuclei. Furthermore, the *P. armatulus* specimens were half as long as the *S. glanis* (SL: 11–12 cm versus 23–31 cm), yielding the effects already mentioned above.

Note here also that different temperatures were used during tests (recent study, 15°C vs. 25°C; Papes and Ladich, [26]: 22°C vs. 30°C); this resulted in a 10°C difference between highest and lowest temperature in the current study versus an 8°C difference in the study of Papes and Ladich [26]. Nevertheless, latencies decreased when temperature rose. Papes and Ladich [26] already mentioned that this phenomenon could be an effect involving temperature dependence of spike conduction velocity, of spike shape or synaptic delay.

Wysocki and Ladich [31] showed that the minimum pulse period resolvable by the auditory system was below 1.5 ms. This enables otophysines and osphronemids (labyrinth fishes or gouramis) to process each pulse within a series of intraspecific sounds. Current data, however, do not indicate that the temporal resolution in fishes depends on temperature. Papes and Ladich [26] revealed that the minimum resolvable click period is unaffected by changes in temperature in *P. armatulus*.
